# Stereotactic radiotherapy using a linear accelerator for arteriovenous malformation and arteriovenous fistula identification using 320-row angiography-computed tomography images, case series and a single-center experience

**DOI:** 10.20407/fmj.2025-040

**Published:** 2026-05-14

**Authors:** Fumitaka Ito, Yasunori Saito, Takuro Uchida, Kazuya Takahashi, Kazuhiro Murayama, Sinya Hayashi, Haruka Uezono

**Affiliations:** 1 Department of Radiation Oncology, Fujita Health University, School of Medicine, Toyoake, Aichi, Japan; 2 Division of Radiology, Fujita Health University Hospital, Toyoake, Aichi, Japan; 3 Department of Radiology, Fujita Health University, School of Medicine, Toyoake, Aichi, Japan

**Keywords:** Stereotactic radiotherapy, Stereotactic radiosurgery, Image fusion, 320-row computed tomography angiography, Linear accelerator

## Abstract

**Objective:**

Stereotactic radiotherapy (SRT) and stereotactic radiosurgery (SRS) are useful treatments for arteriovenous malformations (AVMs) and arteriovenous fistulas (AVFs). In this study, 320-row four-dimensional (4D) computed tomography angiography (CTA) was used to determine the irradiation range before SRT/SRS, and the treatment effect was retrospectively evaluated.

**Methods:**

Five patients with an AVM and two with an AVF who underwent 4D CTA were included. Treatment was planned and performed based on the 320-row CTA images. Treatment efficacy was evaluated using magnetic resonance imaging and CTA.

**Results:**

Shrinkage was observed in four of the five AVMs and both AVFs. Additional irradiation was performed in one AVM patient and one AVF patient. One AVM patient experienced seizures 6 years after treatment. No other adverse neurological events or cerebral hemorrhages occurred.

**Conclusion:**

320-row 4D CTA image fusion was useful for lesion identification in determining radiation treatment plans for AVMs and AVFs. It also was able to demonstrate post-treatment shrinkage and disappearance of lesions.

## Introduction

Supratentorial arteriovenous malformations (AVMs) and dural arteriovenous fistulas (AVFs) are congenital vascular malformations. Dural AVFs associated with cortical venous reflux and those causing intracranial bleeding and/or intolerable symptoms should be treated.^1^ AVMs put patients at risk for cerebral hemorrhage, and treatment is indicated.^2^ The goal of AVM treatment is complete removal or obliteration to reduce the risk of hemorrhage while minimizing the risk of treatment-related complications. These factors depend on the size and location of the lesion. Surgery and endovascular obliteration are treatment options for both types of malformation, as is stereotactic radiosurgery (SRS), which is generally reserved for inoperable lesions.^3–6^ However, treatment of large AVMs (focal volume ≥12 cm^3^) with a single session of SRS alone generally produces poor results.^6^ Volume-staged SRS may be considered for large AVMs, but requires angiography and multiple SRS treatment session to thoroughly treat the entire AVM.^7^

In general, fused angiography images are used to determine the irradiation range with Gamma Knife (Elekta, Stockholm, Sweden) SRS. In one study of SRS workflow for AVM treatment, the mean time for the combined processes of frame application, patient transportation, and frame removal was 132 minutes.^8^ When the irradiation volume for an AVM is large, fractionated irradiation can decrease the late effects of radiation damage.^9^ Although conventional Gamma Knife SRS requires placement of a fixed head frame, which is somewhat invasive, AVM treatment is also possible using stereotactic radiotherapy (SRT) with a linear accelerator (LINAC).^10^ With LINAC, daily divided SRT is possible without placing a head frame on the patient. Compared with frame-based SRS, treatment times are shorter with LINAC SRT. In addition, LINAC SRT can reduce exposure of normal brain tissue to radiation.

Conventionally, digital subtraction angiography (DSA) is used to identify the AVM nidus. DSA provides excellent resolution, which allows differentiation of the nidus from the feeding arteries and draining veins. However, DSA images are two-dimensional and, therefore, not useful for delineating the lesion in three-dimensional (3D) radiation planning. In addition, DSA images cannot provide useful fusion images for LINAC SRT.

Therefore, 3D imaging modalities, such as computed tomography angiography (CTA) and magnetic resonance angiography (MRA) are used for planning LINAC SRT of AVMs. Whereas CTA and MRA provide a view of the vascular tree with excellent 3D localization, a drawback of the standard “static” CTA/MRA is that the images represent a snapshot of the blood flow through the AVM and draining veins at a pre-determined acquisition time. This snapshot is unlikely to be the optimal time-point for viewing the nidus, which can make differentiation of the nidus from the surrounding angio-architecture challenging.^11^

Four-dimensional (4D) CTA provides not only the anatomical information of conventional 3D CTA, but also detailed visualization of blood flow dynamics. However, disadvantages include longer imaging time and higher radiation exposure. 4D CTA obtained using a 320-row detector provides more detailed brain images than conventional 3D CTA.^12^ Therefore, using 4D CTA in imaging of AVMs and AVFs before SRT or SRS should enable more accurate identification and delineation of the nidus and fistula. In this study, we report our experience with using 320-row 4D CTA in the SRT or SRS of seven patients with an AVM or AVF and evaluate the treatment effects.

## Materials and methods

This study was approved by the ethics review board (research numbers: HM-16-372, HM-13-077) and was performed in accordance with the tenets of the 1964 Declaration of Helsinki and subsequent revisions. All patients provided written informed consent.

### Patients

**Figure 1:**
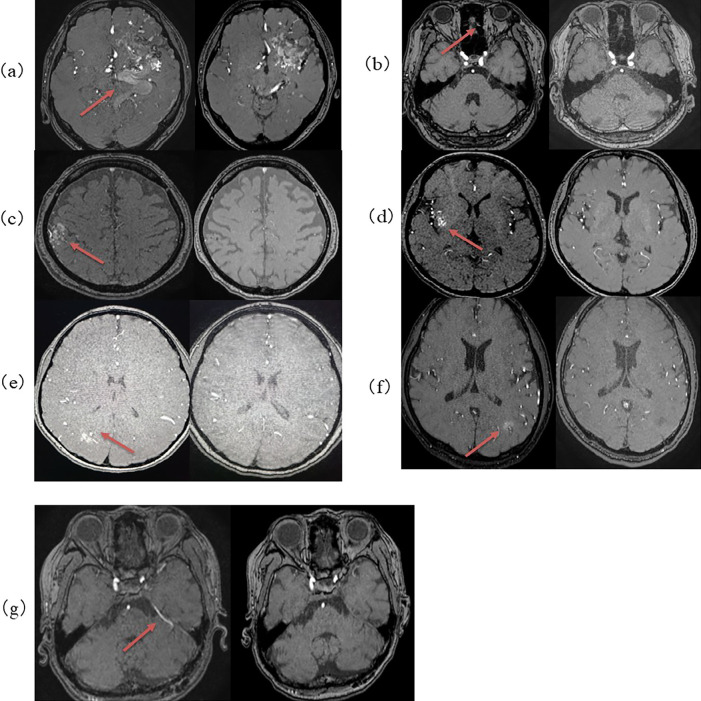
Magnetic resonance angiography images before and after irradiation. a) Case 1. b) Case 2. c) Case 3. d) Case 4. e) Case 5. f) Case 6. g) Case 7. The arrows indicate the arteriovenous malformation or fistula. Left images were obtained before treatment, and right images after.

Seven patients (five with a supratentorial AVM and two with an AVF) underwent SRS or SRT based on fused 320-row 4D CTA from 2013 to 2019 at Fujita Health University Hospital. Median patient age was 50 years (range, 9–75). Mean follow-up was 81 months (range, 43–111). Treatment was defined as SRS when a single irradiation session was performed; multiple session treatment was defined as SRT. Two patients underwent additional treatment after initial SRT: case 1 (AVM) underwent additional SRT and case 2 (AVF) underwent additional SRS. Among the five AVM patients, four underwent SRS or SRT alone without transcatheter arterial embolization (TAE) before the procedure. Both AVF patients underwent pretreatment TAE. Treatment and lesion details are shown in Table 1. MRA imaging before treatment is shown in Figure 1.

### Image acquisition

All patients underwent 320-row 4D CTA and magnetic resonance imaging (MRI). Volume-rendered computed tomography was performed using the Infinix-I angiography device (Canon Medical, Nasu, Japan) and Aquillion ONE hybrid CT system (Canon Medical). On CTA, an image that best identified the nidus or fistula in the arterial phase was identified. The CT imaging conditions were as follows: tube voltage, 80 kV; tube current, 150 mA, rotation speed, 0.275 s/rot, and field of view, 240.0 mm. Radiation treatment planning CT was then performed using the Aquillion LB system (Canon Medical) with mask fixation and slice thickness of 1 mm. The 2015 national diagnostic reference levels (DRLs) of the Japan Network for Research and Information on Medical Exposure were applied to prevent excessive radiation exposure. We also confirmed that doses did not exceed the standardized computed tomography dose index volume limit, (<60 mGy).

**Figure 2:**
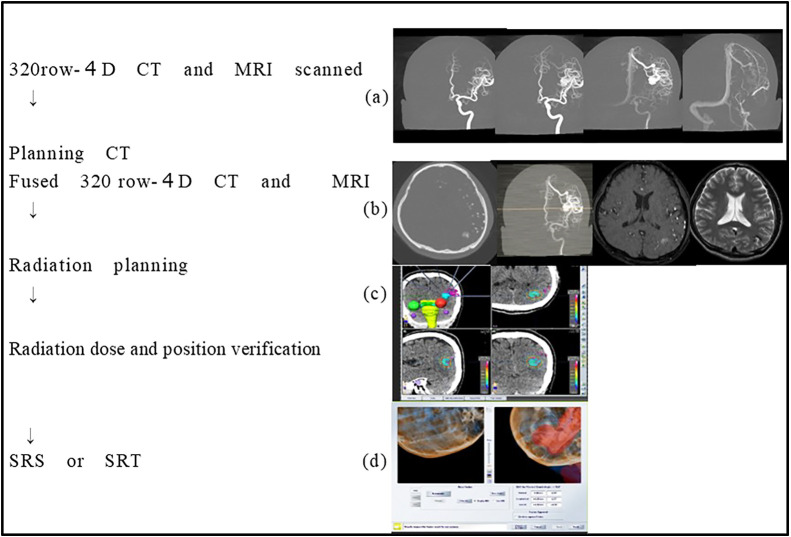
Treatment flowchart. a) 320-row four-dimensional computed tomography angiography—a catheter was inserted, and a time phase that allows the lesion to be identified was applied. b) Image extraction and fusion of the computed tomography angiography and magnetic resonance images. c) Radiation treatment planning was based on these images. d) Stereotactic radiosurgery or radiotherapy was then performed.

4D CTA images, T2-weighted images, and radiation treatment planning CT images acquired before treatment were transferred to the treatment planning system as fusion images. iPlan RT Image and BrainSCAN software (BrainLAB, Heimstetten, Germany) were applied to the fusion images to show the time phase that best showed the nidus on 4D CTA. An image acquisition flowchart is shown in Figure 2.

### Radiation planning

Gross tumor volume and clinical target volume were defined as the nidus or fistula. Niduses were identified on fused T2-weighted images and 4D CTA images, whereas fistulas were identified on 4D CTA images. The planning target volume included an additional 2 mm around the gross and clinical target volumes.

Large AVMs exceeding 10 cm^3^ were treated using SRT to lower the radiation dose applied to the normal brain and avoid radiation complications.^13^ For smaller ones, Gamma Knife SRS was performed.^5,10^ AVFs were treated using SRS.^5^ Based on previous studies, the initial treatment plan was to administer a minimum dose of approximately 20 Gy to the target.^14^ In the two patients who underwent additional treatment, the dose was reduced to account for overlap with the previous irradiation area (Table 1).

### Radiation therapy

Radiation dosimetry was verified for all lesions as the isocenter dose prior to irradiation. All irradiation was performed with isocenter positional error <1 mm in adaptive image-guided radiotherapy using an ExacTrac image matching device (BrainLAB). LINAC SRS/SRT was performed using Novalis Tx (BrainLAB). Non-coplanar irradiation and head shell fixation were applied in all cases.

### Follow-up

Treatment effects were evaluated using MRI and MRA at least once a year. The end points were the presence or absence of bleeding and the presence or absence of lesion shrinkage. If treatment was not sufficiently effective, 4D CTA was performed at the 3 year follow-up. The decision to apply additional treatment was then made based on the findings. Adverse events were evaluated according to the Common Terminology Criteria for Adverse Events version 4.0.^15^

## Results

### Image acquisition

The nidus or fistula was successfully identified using fused T2-weighted and 4D CTA images in all seven patients.

### Radiation therapy

The radiation plan localized to the lesion was created based on the 4D CTA and magnetic resonance images. Irradiation was performed with an inter- and intra-fractional set-up error <1 mm in all cases. Head shell fixation was applied in all cases. The irradiation time was under 40 minutes in all cases.

### Treatment effect

**Figure 3:**
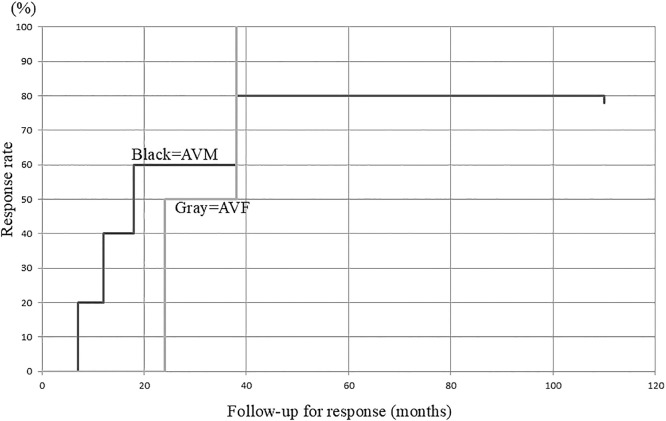
Treatment response rates over time.

Additional irradiation was performed after the initial treatment in one AVM patient and one AVF patient. At the 5-year follow-up, lesion shrinkage had occurred in four of the five AVM patients and both AVF patients (Figure 3). In case 1, the AVM was large, and partial shrinkage was seen on follow-up images, but nidus occlusion was not achieved. MRA images after treatment are shown in Figure 1. No AVM or AVF bleeding events occurred during follow-up.

### Complications

In case 1, the patient’s seizures improved after the first round of SRT; however, anticonvulsant medication was continued. In case 6, cerebral edema developed around the AVM 3 years after irradiation; seizures developed 6 years after irradiation and an anticonvulsant was initiated. No hemorrhagic changes were observed on post-treatment imaging.

## Discussion

Treatment planning systems for SRT now include 320-row 4D CTA images.^12^ In our study of seven patients, 320-row 4D CTA images successfully identified the specific target lesion (AVM nidus, AVF fistula) before treatment, and LINAC SRT or SRS was subsequently administered.

Unlike Gamma Knife SRS, which requires application of a frame affixed to the patient’s head, our patients underwent SRS or SRT using a mask that is easy to put on and take off. By performing 320-row 4D CTA and MRI before treatment, radiotherapy time in our sessions was shorter than that reported in a previous Gamma Knife study.^8^ Follow-up was performed for more than 5 years after treatment, and we found that both SRS and SRT were effective treatments for AVMs and for AVFs which had been previously embolized.

**Figure 4:**
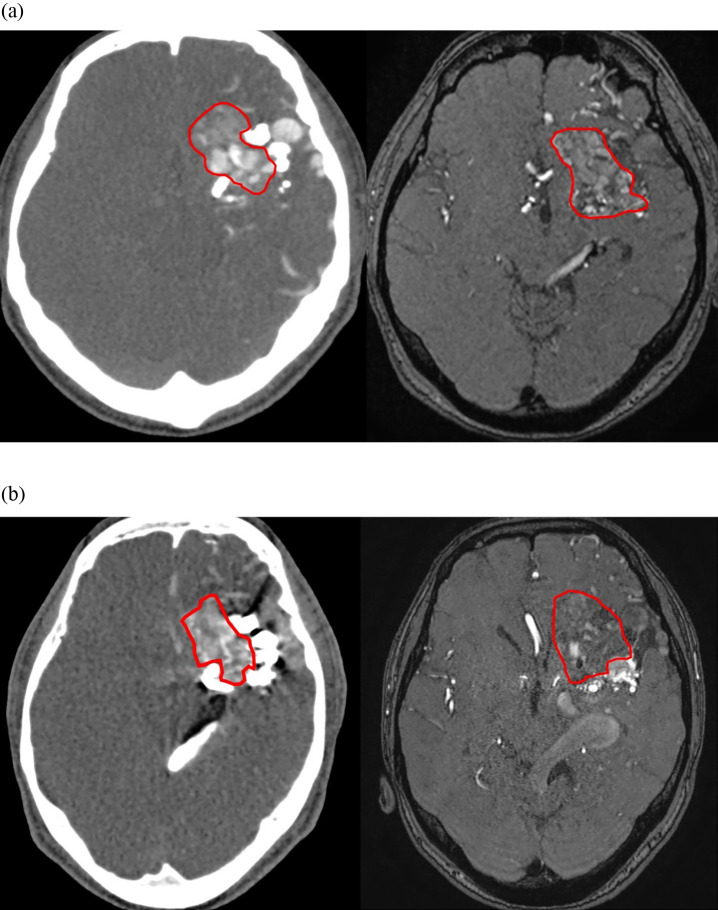
Fused four-dimensional computed tomography angiography imaging in case 1 reduced the clincal target volume compared with magnetic resonance angiography (MRA)-only fusion. a) In the first treatment, the planning target volumes for computed tomography angiography and magnetic resonance angiography were 20.54 and 32.99 cc, respectively. b) In the second treatment, the corresponding volumes were 15.94 and 30.54 cc, respectively. In both panels, the same slices are shown.

In case 1, who harbored an AVM exceeding 22 cm^3^ in size, the initial treatment was only partially effective, as obliteration was not achieved. This is fairly common when treating large AVMs using volume-staged SRS.^7^ Fused 4D CTA imaging was found to reduce the planning target volume compared with MRA-only fusion (Figure 4).

In particular, for large AVMs (>10 cm^3^), the radiation dose to the surrounding normal brain increases, making it difficult to deliver a sufficient dose to avoid radiation complications. When aiming to reduce the dose to the brain during treatment planning, dose-graded radiosurgery using fractionated irradiation was performed.^16^ For smaller AVMs, such as those in cases 5 and 6, SRS was performed in accordance with the treatment method used for Gamma Knife SRS.^5,10^ For AVFs, SRS was performed as in previous studies.^5^ However, in the first treatment of case 2, the AVF was located close to the patient’s eyes (Figure 1), making it difficult to administer a high dose with SRS, so SRT was performed instead.

After treatment, one AVM patient developed seizures 6 years after treatment. The other six experienced no neurological adverse events or cerebral hemorrhage during follow up. Furthermore, no hemorrhagic changes on imaging were observed. Based on these results, LINAC SRS and SRT using 320-row 4D CTA can be considered a viable treatment option for AVMs and AVFs, particularly when the therapeutic effect of TAE has been insufficient

Compared with identifying the nidus of an AVM, identifying the precise site of the fistula in an AVF can be difficult. However, it is imperative to deliver radiation to this site to achieve fistula occlusion. The data from our study show that 320-row 4D CTA is effective in identifying fistulas; moreover, it can confirm fistula occlusion after treatment.

According to a previous study, fusion treatment planning using 320-row 4D CTA has the potential to reduce the irradiated volume compared with planning performed using conventional two-dimensional angiography.^11^ AVM size reduction can also be evaluated more objectively on follow-up imaging using 4D CTA.

The effect of reducing the treatment volume has been confirmed after observing treatment progress. With the exception of one patient, no adverse neurological events occurred. Therefore, we conclude that radiation damage to normal brain was avoided.

LINAC SRS/SRT using a fixed mask is less invasive and easier to apply than frame-based Gamma Knife SRS. LINAC SRT had dose prescription variations, unlike SRS (single fraction). Given this benefit, our study suggests that LINAC may provide a more effective treatment for large AVMs (>20 cm^3^).

By comparing the bones, it was possible to perform irradiation with an accuracy of less than 1 mm. Based on the treatment results after SRT, lesion identification using 320-row 4D CTA fused imaging was accurate, and the position fixation accuracy during SRT was judged to be excellent.

Because a mask was used and not a head frame, treatment times were less than 40 minutes, which is considerably shorter than those associated with conventional SRS.^8^ This makes it possible to perform SRS/SRT treatments without requiring anesthesia or sedation. Avoiding sedation-related adverse events is an advantage. In addition, avoiding frame-based treatment is beneficial and safer when treating children.

A wider irradiation area results in a more widespread area of brain necrosis, increasing the risk of neurological damage; the area where radiation-induced secondary cancer may occur is wider as well.^17^ In our study, by using 320-row 4D CTA, the irradiation area was smaller, which is beneficial for young AVM patients.

This study is limited by its retrospective single-center design and small sample size. One large AVM (>20 cm^3^) required two treatments, and, although it was partially reduced, complete occlusion was not achieved.

## Conclusion

This study showed that 320-row 4D CTA image fusion was useful for lesion identification when determining radiation treatment plans for AVMs and AVFs. It was also able to demonstrate lesion shrinkage and disappearance after treatment.

## Figures and Tables

**Table 1:** Treatment and lesion details.

	Treatment	Dose /Fractionations	Definition of Prescription	Irradiation method	Disease	Lesion	Volume (cc)	Pretraeatment
Case1	SRT	31.5Gy/9Fr	D85	Conformal beam	AVM	Lt. Frontal	26.54	TAE 3times
Case1	SRT additional	30Gy/5Fr	D95	Dynamic conformal arc	AVM	Lt. Frontal	15.95	TAE 3times
Case2	SRT	25Gy/5Fr	D95	Dynamic conformal arc	AVF	Lt. Frontal	4.46	TAE 3times
Case2	SRS additional	15Gy/1Fr	D95	Dynamic conformal arc	AVF	Lt. Frontal	0.42	TAE 3times
Case3	SRT	30Gy/5Fr	D90	Dynamic conformal arc	AVM	Rt. Parietal	2.47	none
Case4	SRT	30Gy/5Fr	D95	Dynamic conformal arc	AVM	Rt. Basal nucleus	2.07	none
Case5	SRS	21Gy/1Fr	D95	Dynamic conformal arc	AVM	Rt. Occipital	0.44	none
Case6	SRS	18Gy/1Fr	minimun dose	Dynamic conformal arc	AVM	Lt. Parietal	1.2	none
Case7	SRS	20Gy/1Fr	D95	Dynamic conformal arc	AVF	Lt. Parietal	1.33	TAE 3times

## References

[1] Elhammady MS, Ambekar S, Heros RC. Epidemiology, clinical presentation, diagnostic evaluation, and prognosis of cerebral dural arteriovenous fistulas. Clin Neurol; 2017 143: 99–105. 10.1016/B978-0-444-63640-9.00009-6 28552162

[2] Karlsson B, Jokura H, Yang HC, Yamamoto M, Martinez R, Kawagishi J, Guo WY, Beute G, Chung WY, Söderman M, Yeo TT. Clinical outcome following cerebral AVM hemorrhage. Acta Neurochir 2020; 162: 1759–1766. 32385636 10.1007/s00701-020-04380-z

[3] Rutledge C, Cooke DL, Hetts SW, Abla AA. Brain arteriovenous malformations. Handb Clin Neurol 2021; 176: 171–178. 33272394 10.1016/B978-0-444-64034-5.00020-1

[4] Cenzato M, Boeris D, Piparo M, Fratianni A, Piano MA, Dones F, M Crisà F, D’Aliberti G. Complications in AVM surgery. Acta Neurochir Suppl 2021; 132: 77–81. 33973032 10.1007/978-3-030-63453-7_11

[5] Gross BA, Du R. Diagnosis and treatment of vascular malformations of the brain. Curr Treat Options Neurol 2014; 16: 279. 24318447 10.1007/s11940-013-0279-9

[6] Ilyas A, Ding D, Hixson HR, Xu Z, Starke RM, Sheehan JP. Volume-staged stereotactic radiosurgery for large intracranial arteriovenous malformations. J Clin Neurosci 2017; 43: 202–207. 28495425 10.1016/j.jocn.2017.04.020

[7] Pollock BE, Link MJ, Stafford SL, Lanzino G, Garces YI, Foote RL. Volume-staged stereotactic radiosurgery for intracranial arteriovenous malformations: outcomes based on an 18-year experience. Neurosurgery 2017; 80: 543–550. 28362923 10.1093/neuros/nyw107

[8] Dabus G, Kotecha R, Linfante I, Wieczorek DJ, Gutierrez AN, Candela JG, McDermott MW. Analysis of potential time saving in brain arteriovenous malformation stereotactic radiosurgery planning using a new software platform. Med Dosim 2022; 47: 38–42. 34481717 10.1016/j.meddos.2021.07.004

[9] Milano MT, Grimm J, Niemierko A, Soltys SG, Moiseenko V, Redmond KJ, Yorke E, Sahgal A, Xue J, Mahadevan A, Muacevic A, Marks LB, Kleinberg LR. Single- and Multifraction stereotactic radiosurgery dose/volume tolerances of the brain. Int J Radiat Oncol Biol Phys 2021; 110: 68–86. 32921513 10.1016/j.ijrobp.2020.08.013 PMC9387178

[10] Orio P, Stelzer KJ, Goodkin R, Douglas JG. Treatment of arteriovenous malformations with linear accelerator–based radiosurgery compared with Gamma Knife surgery. J Neurosurg 2006; 105: 58–63. 18503331 10.3171/sup.2006.105.7.58

[11] Haridass A, Maclean J, Chakraborty S, Sinclair J, Szanto J, Iancu D, Malone S. Dynamic CT angiography for cyberknife radiosurgery planning of intracranial arteriovenous malformations: a technical/feasibility report. Radiol Oncol 2015; 49: 192–199. 26029032 10.1515/raon-2015-0006 PMC4387997

[12] Hayakawa M, Tanaka T, Sadato A, Adachi K, Ito K, Hattori N, Omi T, Oheda M, Katada K, Murayama K, Kato Y, Hirose Y. Detection of pulsation in unruptured cerebral aneurysms by ECG-gated 3D-CT angiography (4D-CTA) with 320-row area detector CT (ADCT) and follow-up evaluation results: assessment based on heart rate at the time of scanning. Clin Neuroradiol 2014; 24: 145–150. 23913018 10.1007/s00062-013-0236-8

[13] Treuer H, Kocher M, Hoevels M, Hunsche S, Luyken K, Maarouf M, Voges J, Müller RP, Sturm V. Impact of target point deviations on control and complication probabilities in stereotactic radiosurgery of AVMs and metastases. Radiother Oncol 2006; 81: 25–32. 17005278 10.1016/j.radonc.2006.08.022

[14] Flickinger JC, Pollock BE, Kondziolka D, Lunsford LD. A dose-response analysis of arteriovenous malformation obliteration after radiosurgery. Int J Radiat Oncol 1996; 36: 873–879. 10.1016/s0360-3016(96)00316-1 8960516

[15] Hay JL, Atkinson TM, Reeve BB, et al. Cognitive interviewing of the US National Cancer Institute’s patient-reported outcomes version of the common terminology criteria for adverse events (PRO-CTCAE). Qual Life Res 2014; 23: 257–269. 23868457 10.1007/s11136-013-0470-1 PMC3896507

[16] Mohan A, Tiwari S, Pareek P, Fernandes A, Santhyavu S, Kombathula SH, Choubisa M, Gayen S, Irfad M, Solanki A. Linear Accelerator (LINAC) Radiosurgical Management of Brain Arteriovenous Malformations: An Experience From a Tertiary Care Center. Cureus 2024; 16: e76232. 39845248 10.7759/cureus.76232 PMC11751660

[17] Yamanaka R, Hayano A. Radiation-induced sarcomas of the central nervous system: a systematic review. World Neurosurg 2017; 98: 818-28.e7. 10.1016/j.wneu.2016.11.008 27838424

